# The pendular regulation model: a transdiagnostic approach to emotional regulation

**DOI:** 10.3389/fpsyg.2026.1813038

**Published:** 2026-10-22

**Authors:** Andres F. Mambuca

**Affiliations:** Mental Health Institute of Florida, Pembroke Pines, FL, United States

**Keywords:** identity coherence, neurovisceral integration, nonlinear dynamics, pendular regulation model, predictive processing, transdiagnostic regulation

## Abstract

Contemporary psychiatric classification systems face persistent concerns regarding diagnostic heterogeneity, comorbidity, and limited mechanistic precision. Although dimensional and transdiagnostic initiatives—including HiTOP, RDoC, and network models—have advanced the field, a unifying regulatory architecture integrating nonlinear dynamics, hierarchical predictive processing, stress physiology, and identity stabilization remains underdeveloped. The present article introduces the Pendular Regulation Model (PRM), a neurodynamic transdiagnostic framework conceptualizing psychological functioning as bounded oscillatory regulation within hierarchical predictive systems embedded in neurovisceral and stress-responsive networks. Within this model, psychopathology reflects maladaptive stabilization patterns characterized by regulatory constriction or destabilization. Identity coherence is proposed as an emergent property of repeated regulatory trajectories over time. The manuscript outlines a structured empirical agenda designed to evaluate the model’s longitudinal predictive capacity using established quantitative methods. Explicit falsifiability criteria are specified. If empirically supported, the PRM may offer an integrative regulatory framework capable of clarifying cross-diagnostic variability and informing treatment responsiveness.

## Introduction

Psychiatric nosology has advanced substantially in reliability over recent decades; however, concerns regarding construct validity, diagnostic heterogeneity, and excessive comorbidity persist (Hyman, 2010; Kendler, 2016). Individuals meeting criteria for the same categorical diagnosis frequently display divergent symptom profiles, trajectories, and treatment responses. Such variability challenges assumptions of discrete underlying disease entities and motivates the continued development of dimensional and transdiagnostic frameworks.

In response, multiple alternative models have emerged. The Hierarchical Taxonomy of Psychopathology (HiTOP) reorganizes diagnostic phenomena into empirically derived spectra reflecting shared covariance structures (Kotov et al., 2017). The Research Domain Criteria (RDoC) initiative seeks to ground psychopathology within neurobehavioral systems that cut across traditional diagnostic categories (Insel et al., 2010). Network approaches conceptualize mental disorders as dynamic systems of interacting symptoms rather than latent diseases (Borsboom, 2017). Process-based therapy emphasizes modifiable mechanisms operating across diagnostic boundaries (Hofmann and Hayes, 2019; Hayes et al., 2020). Collectively, these models represent significant progress toward dimensional and mechanistic understanding.

Nevertheless, current frameworks tend to emphasize either structural covariance (HiTOP), neurofunctional systems (RDoC), symptom interaction dynamics (network theory), or therapeutic mechanisms (process-based approaches) without fully articulating a unified regulatory architecture integrating nonlinear system dynamics, hierarchical predictive inference, stress physiology, and long-term identity consolidation within a single explanatory scaffold.

Psychological functioning unfolds across multiple interacting timescales. Emotional states fluctuate over minutes or hours, cognitive priors update across contexts, and identity consolidates across developmental epochs. Simultaneously, regulatory processes operate within neurovisceral systems that link prefrontal control networks with autonomic modulation (Thayer and Lane, 2000; Beauchaine and Thayer, 2015), while predictive processing mechanisms govern precision-weighted belief updating (Friston, 2010; Clark, 2016). An integrative account must therefore accommodate oscillatory variability, hierarchical inference, physiological regulation, and temporal stabilization without reducing psychopathology to static categorical entities.

The present article introduces the Pendular Regulation Model (PRM), a neurodynamic transdiagnostic framework conceptualizing psychological functioning as bounded oscillatory regulation within hierarchical predictive systems embedded in stress-responsive neurovisceral networks. Within PRM, adaptive functioning reflects preserved oscillatory tolerance within regulatory bounds, whereas psychopathology reflects maladaptive stabilization patterns characterized by regulatory constriction or destabilization. Identity coherence is proposed as an emergent property of repeated regulatory trajectories over time.

*The primary objective of this manuscript is threefold*: (1) to formally articulate the theoretical foundations of PRM within nonlinear dynamical systems, predictive processing, and neurovisceral integration frameworks; (2) to operationalize key regulatory constructs in measurable terms; and (3) to outline a structured, falsifiable empirical research program designed to test incremental predictive validity beyond established constructs. By situating PRM as empirically vulnerable and methodologically tractable, the model is presented not as a replacement for existing frameworks, but as a potential integrative scaffold subject to systematic evaluation.

*Clinical and Research Positioning of PRM*. PRM is not proposed as an alternative diagnostic taxonomy or as a replacement for existing clinical frameworks. Rather, its potential contribution lies in characterizing temporal and regulatory dynamics that may underlie heterogeneous symptom presentations across diagnostic categories. In clinical assessment, the model could be used to describe whether an individual’s functioning is characterized predominantly by regulatory constriction, destabilization, or preserved oscillatory tolerance. At the mechanistic level, PRM generates hypotheses regarding the interaction among updating flexibility, precision calibration, stress physiology, and recovery dynamics. In treatment research, the model may provide a framework for examining whether interventions alter regulatory range, recovery velocity, or attractor stability over time. These applications remain hypotheses for empirical testing rather than established clinical uses.

### The nonlinear regulatory architecture of psychological functioning

Complex adaptive systems are characterized by nonlinear feedback, multistability, and emergent organization (Kelso, 1995; van der Maas et al., 2006). In such systems, identical inputs may produce divergent outputs depending on system history, internal configuration, and parameter thresholds. Psychological functioning exhibits similar properties. Emotional responses, cognitive appraisals, physiological activation, and behavioral tendencies interact recursively across time, forming self-reinforcing patterns that resist or amplify perturbations.

Within nonlinear systems theory, attractor states represent relatively stable configurations toward which systems converge. Attractor basins vary in depth, width, and permeability. Deep basins resist perturbation; shallow basins permit frequent transitions. PRM adopts this dynamical language cautiously, not as metaphor alone but as a structural framework for modeling regulatory behavior across levels of organization.

Regulation is conceptualized as bounded oscillatory movement within attractor geometry. Oscillation refers to patterned fluctuation rather than instability. Adaptive systems exhibit preserved variability within integrative thresholds. Too little variability reflects rigidity; excessive variability reflects destabilization. This distinction aligns with empirical findings that both emotional suppression and affective volatility predict dysfunction (Gross, 2015). PRM distinguishes between two maladaptive regulatory geometries:

### Regulatory constriction

Constriction corresponds to excessively deep and narrow attractor basins. Such systems resist updating even when environmental contingencies change. Hyper-stable rumination, persistent threat anticipation, compulsive avoidance, and entrenched self-belief may represent constricted geometries. Within predictive frameworks, constriction may reflect hyper-precise high-level priors that override corrective sensory input (Friston, 2010).

### Regulatory destabilization

Destabilization corresponds to shallow or poorly defined basins lacking containment. Minor perturbations produce disproportionate shifts. Emotional volatility, rapid state-switching, and fragmented self-coherence may exemplify destabilized geometry. Within predictive models, destabilization may reflect insufficient hierarchical integration or unstable precision calibration.

Importantly, PRM differentiates emotional intensity from regulatory instability. High arousal may remain adaptive when integrated within bounded geometry. Conversely, low-intensity fluctuation may destabilize when hierarchical containment fails.

Phase transition phenomena further characterize nonlinear systems. Gradual accumulation of stressors may precipitate abrupt reorganization once thresholds are exceeded. Clinically, individuals often report sudden symptom escalation following prolonged subclinical strain. Such tipping-point dynamics align with nonlinear threshold behavior observed in complex systems (Pincus and Metten, 2010).

Multistability provides additional explanatory depth. Systems may possess multiple potential attractors. Environmental triggers or internal perturbations determine which basin dominates. This framework accounts for heterogeneity in response to similar stress exposure. Rather than invoking latent disease entities, PRM conceptualizes symptom expression as stabilization within particular regulatory geometries.

*Clinical Expression Across Regulatory Geometries*. These regulatory geometries are not intended to map one-to-one onto diagnostic categories. Similar symptom presentations may arise from different regulatory configurations, while different disorders may share comparable underlying regulatory dynamics. For example, persistent anxiety may involve constricted threat-related priors and reduced updating, whereas affective lability may reflect insufficient containment and rapid state transition. Accordingly, PRM generates the testable prediction that clinically similar symptoms may differ in their temporal dynamics, recovery patterns, and physiological correlates. Longitudinal assessment would therefore be necessary to distinguish regulatory geometry from symptom intensity alone.

### Hierarchical predictive processing and precision calibration

Predictive processing models conceptualize the brain as a hierarchical generative model that minimizes prediction error through recursive updating (Clark, 2013, 2016; Friston, 2010). At each hierarchical level, predictions descend while prediction errors ascend. Crucially, prediction errors are weighted by precision—the estimated reliability of incoming signals.

Precision calibration determines system flexibility. Excessively precise priors inhibit updating, producing rigidity. Insufficient precision produces volatility and overcorrection. Adaptive regulation requires calibrated balance between stability and flexibility.

Within PRM, oscillatory tolerance reflects macro-level expression of calibrated precision assignment. Constriction may correspond to hyper-precision at higher hierarchical levels, such that high-level beliefs dominate perception. For example, persistent threat expectancy in anxiety may reflect excessive precision assigned to threat-related priors (Peters et al., 2017). Corrective environmental feedback fails to recalibrate the system.

Conversely, destabilization may reflect unstable precision weighting across levels. Inconsistent top-down modulation may permit lower-level signals to drive excessive updating, resulting in volatility.

Prefrontal–limbic integration plays a central role in this calibration. The prefrontal cortex supports contextual inference and inhibitory modulation, while limbic structures encode salience and affective intensity (Miller and Cohen, 2001). Adaptive regulation depends on coordinated bidirectional communication across these systems. Dysregulated connectivity may deepen constricted basins or weaken containment in destabilized states.

The free energy principle extends this framework by proposing that biological systems maintain structural integrity through minimizing surprise across time (Friston, 2010). Constricted states may reduce immediate uncertainty by rigidly stabilizing priors, whereas destabilized states may reflect failure to maintain coherent generative structure. PRM situates oscillatory tolerance within this balance: adaptive systems tolerate bounded uncertainty while preserving integrative coherence.

Importantly, PRM does not claim direct clinical measurement of precision parameters. Rather, behavioral and psychometric proxies—such as updating flexibility, intolerance of uncertainty, and recovery velocity—may indirectly index underlying hierarchical calibration.

### Neurovisceral integration and stress physiology

The neurovisceral integration model proposes that adaptive emotional regulation depends on functional integration between prefrontal cortical systems and autonomic output (Thayer and Lane, 2000). Vagal tone, often indexed by heart rate variability (HRV), reflects regulatory capacity and inhibitory control. Reduced HRV has been associated with anxiety, depression, and impaired flexibility (Beauchaine and Thayer, 2015).

Within PRM, HRV is conceptualized as a peripheral correlate of oscillatory tolerance. Systems exhibiting preserved oscillatory range demonstrate dynamic variability coupled with efficient recovery following stress. Chronic sympathetic dominance may narrow regulatory range, deepening defensive attractor basins.

Allostatic load research further supports this integration. Chronic stress exposure reshapes neural architecture through glucocorticoid-mediated modulation of prefrontal and hippocampal systems (McEwen and Morrison, 2013). Persistent stress may impair top-down regulation, reduce containment capacity and increase vulnerability to destabilization.

PRM differentiates between adaptive recalibration and maladaptive narrowing. In dangerous contexts, defensive stabilization may be protective. Pathology emerges when environmental contingencies shift but regulatory geometry remains rigidly stabilized or chronically destabilized.

Integrating predictive processing, nonlinear dynamics, and neurovisceral models situates PRM as multi-level. Regulation spans cortical inference, limbic salience encoding, and autonomic modulation. Oscillatory tolerance reflects coordinated integration across these levels.

### Identity as emergent regulatory stabilization

Identity has historically been conceptualized through multiple theoretical lenses, ranging from trait-based models to narrative and constructivist approaches. Contemporary scholarship increasingly converges on the view that identity is neither a static essence nor a purely narrative artifact, but an emergent property of distributed neural and psychological processes (Gallagher, 2013; Metzinger, 2003). PRM extends its regulatory architecture into this domain by conceptualizing identity coherence as the long-term stabilization of repeated regulatory trajectories within hierarchical predictive systems.

From a predictive processing standpoint, identity may correspond to high-level priors embedded within hierarchical generative models. These priors organize perception, memory consolidation, affective interpretation, and future expectation. Over time, recurrent oscillatory patterns consolidate into stable high-level expectations about the self and the world. Identity thus emerges as a stabilized attractor configuration shaped by repeated regulatory cycles.

This perspective reframes identity development as a dynamical process. Repeated exposure to specific relational, environmental, and affective conditions sculpts regulatory geometry. Early attachment disruptions, chronic unpredictability, or persistent threat exposure may deepen defensive attractor basins. These basins gradually consolidate into identity-level beliefs (“I am unsafe,” “Others are unreliable”). Conversely, consistent relational stability may promote adaptive oscillatory tolerance and flexible identity integration. PRM distinguishes among three identity-related geometries.

### Rigid identity stabilization

Excessively precise high-level priors resist updating. Narrative coherence becomes overconsolidated, limiting adaptive recalibration. This rigidity parallels constricted regulatory basins.

### Fragmented identity stabilization

Insufficient consolidation of high-level priors yields unstable self-coherence. Lower-level fluctuations dominate, resulting in inconsistent self-representation. This pattern parallels destabilized regulatory geometry.

### Adaptive identity plasticity

High-level priors remain stable yet permeable. Updating occurs without loss of coherence. This reflects preserved oscillatory tolerance across hierarchical levels. Developmentally, identity consolidation corresponds to iterative integration across regulatory cycles (Habermas and Reese, 2015; McAdams, 2001). PRM situates narrative coherence within the broader regulatory landscape. Identity is not separate from regulation; it is a long-term emergent stabilization of regulatory architecture.

Importantly, this framework avoids metaphysical claims regarding identity localization. Identity coherence likely corresponds to distributed integration across prefrontal, limbic, and interoceptive networks. Empirical studies examining connectivity patterns and autobiographical integration may clarify associations between neural integration and narrative stabilization.

### Comparative positioning within contemporary clinical science

For PRM to contribute meaningfully, it must demonstrate incremental theoretical clarity without overstating novelty. The model is therefore positioned as integrative rather than adversarial.

### Relation to emotion regulation science

Emotion regulation research has identified strategies such as cognitive reappraisal and suppression as central to adaptive functioning (Gross, 2015). PRM situates these strategies within regulatory geometry. Reappraisal may reshape basin depth by altering high-level priors; suppression may temporarily dampen oscillatory amplitude without modifying basin structure. PRM thus provides a structural context for strategy deployment.

### Relation to psychological flexibility

Psychological flexibility is a well-supported transdiagnostic mechanism (Hayes et al., 2020). However, flexibility primarily describes behavioral openness and experiential acceptance. PRM seeks to model the structural constraints within which flexibility operates. Two individuals may exhibit similar flexibility scores yet differ in basin geometry—one constrained but behaviorally compliant, another destabilized but exploratory. Empirical testing must determine whether oscillatory tolerance explains variance beyond flexibility measures.

### Relation to network models

Network theory conceptualizes psychopathology as self-reinforcing symptom systems (Borsboom, 2017). PRM complements this approach by modeling the regulatory landscape in which networks stabilize. Symptom centrality may vary as basin geometry shifts. Integrating network modeling with regulatory range may generate novel hybrid analyses.

### Relation to dimensional taxonomies

HiTOP and RDoC provide dimensional reorganization but do not explicitly articulate oscillatory tolerance. PRM proposes that dimensional spectra reflect underlying regulatory geometries. Mapping future PRM-derived regulatory indices onto dimensional symptom spectra would test compatibility.

### Expanded empirical research program

The scientific viability of the Pendular Regulation Model (PRM) depends on rigorous operationalization and empirical differentiation. Conceptual integration alone does not justify theoretical contribution. The model must demonstrate measurable constructs, incremental predictive validity, mechanistic relevance, and falsifiability. The following staged research program is proposed to systematically evaluate PRM across psychometric, longitudinal, physiological, and interventional domains. In the psychometric stage, indices of oscillatory tolerance, updating flexibility, and recovery velocity would be developed and tested for incremental validity beyond established measures of psychological flexibility and emotion regulation, consistent with feedback-based accounts of self-regulation (Carver and Scheier, 1998). In the longitudinal stage, ecological momentary assessment and intensive longitudinal designs (Stinson et al., 2022; Wrzus and Neubauer, 2023; Hamaker, 2025; Laurenceau et al., 2026) would permit estimation of within-person regulatory range, recovery dynamics, and state transitions, including idiographic and network-based models (Borsboom and Cramer, 2013; Robinaugh et al., 2020; Wright and Woods, 2020). In the physiological stage, HRV recovery profiles (Heiss et al., 2021), interoceptive indices (Critchley and Garfinkel, 2017; Seth, 2013), and markers of cumulative stress burden (McEwen and Stellar, 1993) would be examined as correlates of regulatory geometry, with active inference formulations providing a computational reference for model specification (Friston et al., 2017). In the interventional stage, studies would test whether interventions targeting attentional and regulatory control (Lutz et al., 2008) alter regulatory range and recovery velocity, and whether such changes mediate clinical outcomes.

### Falsifiability and model risk

PRM is empirically vulnerable in several ways:

- If variance in future PRM-derived regulatory indices is fully subsumed by psychological flexibility measures.
- If oscillatory tolerance fails to predict longitudinal recovery trajectories.
- If physiological indices show no systematic association.
- If mediation analyses fail to demonstrate mechanistic contribution.

Scientific credibility requires explicit acknowledgment of these risks. The model is not advanced as established truth but as testable hypothesis.

## Discussion

The Pendular Regulation Model integrates nonlinear dynamics, predictive inference, stress physiology, and identity stabilization into a unified regulatory scaffold. Rather than proposing a new diagnostic taxonomy, PRM articulates a structural geometry underlying transdiagnostic phenomena.

By framing psychopathology as maladaptive stabilization within constricted or destabilized attractor geometries, the model reconciles rigidity and volatility within a single regulatory continuum. Integrating predictive processing situates regulation within hierarchical inference and precision calibration. Incorporating neurovisceral integration grounds regulatory geometry in autonomic physiology. Extending the model to identity situates moment-to-moment oscillation within long-term self-organization. PRM is positioned conservatively. Its value depends on empirical differentiation and incremental validity. If supported, it may provide integrative regulatory geometry organizing dimensional and process-based findings. If unsupported, its limitations will clarify theoretical boundaries.

## Limitations

Several limitations warrant acknowledgment: (1) The model remains conceptual pending operationalization; (2) Overlap with flexibility and regulation constructs must be empirically evaluated; (3) Neurocomputational embedding is inferential rather than directly measurable; (4) Cultural and developmental generalizability require validation; and (5) Physiological correlations represent partial proxies rather than direct measures of geometry. Incremental empirical validation rather than theoretical expansion should guide future development.

## Conclusion

The Pendular Regulation Model proposes that psychological functioning can be conceptualized as bounded oscillatory regulation within hierarchical predictive systems embedded in neurovisceral and stress-regulatory networks. Identity coherence emerges as the long-term stabilization of repeated regulatory trajectories. The model’s value will depend not on its theoretical scope but on whether its constructs can be measured, its predictions tested, and its falsifiability criteria met.

## References

[ref1] Beauchaine T. P. Thayer J. F. (2015). Heart rate variability as a transdiagnostic biomarker of psychopathology. Int. J. Psychophysiol. 98, 338–350. doi: 10.1016/j.ijpsycho.2015.08.004, 26272488

[ref2] Borsboom D. (2017). A network theory of mental disorders. World Psychiatry 16, 5–13. doi: 10.1002/wps.20375, 28127906 PMC5269502

[ref3] Borsboom D. Cramer A. O. J. (2013). Network analysis: an integrative approach to the structure of psychopathology. Annu. Rev. Clin. Psychol. 9, 91–121. doi: 10.1146/annurev-clinpsy-050212-185608, 23537483

[ref4] Carver C. S. Scheier M. F. (1998). On the Self-Regulation of Behavior. Cambridge: Cambridge University Press.

[ref5] Clark A. (2013). Whatever next? Predictive brains, situated agents, and the future of cognitive science. Behav. Brain Sci. 36, 181–204. doi: 10.1017/S0140525X12000477 23663408

[ref6] Clark A. (2016). Surfing Uncertainty: Prediction, Action, and the Embodied Mind. New York, NY: Oxford University Press.

[ref7] Critchley H. D. Garfinkel S. N. (2017). Interoception and emotion. Curr. Opin. Psychol. 17, 7–14. doi: 10.1016/j.copsyc.2017.04.020 28950976

[ref8] Friston K. (2010). The free-energy principle: a unified brain theory? Nat. Rev. Neurosci. 11, 127–138. doi: 10.1038/nrn2787 20068583

[ref9] Friston K. FitzGerald T. Rigoli F. Schwartenbeck P. Pezzulo G. (2017). Active inference: a process theory. Neural Comput. 29, 1–49. doi: 10.1162/NECO_a_00912, 27870614

[ref10] Gallagher S. (2013). The socially extended mind. Cogn. Syst. Res. 25–26, 4–12. doi: 10.1016/j.cogsys.2013.03.008

[ref11] Gross J. J. (2015). Emotion regulation: current status and future prospects. Psychol. Inq. 26, 1–26. doi: 10.1080/1047840x.2014.940781

[ref12] Habermas T. Reese E. (2015). Getting a life takes time: the development of the life story in adolescence, its precursors and consequences. Hum. Dev. 58, 172–201. doi: 10.1159/000437245

[ref13] Hamaker E. L. (2025). Analysis of intensive longitudinal data: putting psychological processes in perspective. Annu. Rev. Clin. Psychol. 21, 379–405. doi: 10.1146/annurev-clinpsy-081423-022947 39914885

[ref14] Hayes S. C. Hofmann S. G. Ciarrochi J. (2020). A process-based approach to psychological diagnosis and treatment: the conceptual and treatment utility of an extended evolutionary meta model. Clin. Psychol. Rev. 82:101908. doi: 10.1016/j.cpr.2020.101908 PMC7680437 32932093

[ref15] Heiss S. Vaschillo B. Vaschillo E. G. Timko C. A. Hormes J. M. (2021). Heart rate variability as a biobehavioral marker of diverse psychopathologies: a review and argument for an “ideal range”. Neurosci. Biobehav. Rev. 121, 144–155. doi: 10.1016/j.neubiorev.2020.12.004, 33309905

[ref16] Hofmann S. G. Hayes S. C. (2019). The future of intervention science: process-based therapy. Clin. Psychol. Sci. 7, 37–50. doi: 10.1177/2167702618772296, 30713811 PMC6350520

[ref17] Hyman S. E. (2010). The diagnosis of mental disorders: the problem of reification. Annu. Rev. Clin. Psychol. 6, 155–179. doi: 10.1146/annurev.clinpsy.3.022806.091532, 17716032

[ref18] Insel T. Cuthbert B. Garvey M. Heinssen R. Pine D. S. Quinn K. . (2010). Research domain criteria (RDoC): toward a new classification framework for research on mental disorders. Am. J. Psychiatry 167, 748–751. doi: 10.1176/appi.ajp.2010.09091379, 20595427

[ref19] Kelso J. A. S. (1995). Dynamic Patterns: The Self-Organization of Brain and Behavior. Cambridge, MA: MIT Press.

[ref20] Kendler K. S. (2016). The nature of psychiatric disorders. World Psychiatry 15, 5–12. doi: 10.1002/wps.20292, 26833596 PMC4780286

[ref21] Kotov R. Krueger R. F. Watson D. Achenbach T. M. Althoff R. R. Bagby R. . (2017). The hierarchical taxonomy of psychopathology (HiTOP): a dimensional alternative to traditional nosologies. J. Abnorm. Psychol. 126, 454–477. doi: 10.1037/abn0000258 28333488

[ref22] Laurenceau J.-P. DiGiovanni A. M. Bolger N. (2026). Intensive longitudinal methods: toward a psychological science of daily life. Annu. Rev. Psychol. 77, 513–541. doi: 10.1146/annurev-psych-040325-025418, 41105835

[ref23] Lutz A. Slagter H. Dunne J. Davidson R. (2008). Attention regulation and monitoring in meditation. Trends Cogn. Sci. 12, 163–169. doi: 10.1016/j.tics.2008.01.005, 18329323 PMC2693206

[ref24] McAdams D. P. (2001). The psychology of life stories. Rev. Gen. Psychol. 5, 100–122. doi: 10.1037/1089-2680.5.2.100

[ref25] McEwen B. S. Morrison J. H. (2013). The brain on stress. Neuron 79, 16–29. doi: 10.1016/j.neuron.2013.06.028, 23849196 PMC3753223

[ref26] McEwen B. S. Stellar E. (1993). Stress and the individual: mechanisms leading to disease. Arch. Intern. Med. 153, 2093–2101. doi: 10.1001/archinte.1993.00410180039004, 8379800

[ref27] Metzinger T. (2003). Being No One: The Self-Model Theory of Subjectivity. Cambridge, MA: MIT Press.

[ref28] Miller G. A. Cohen J. D. (2001). An integrative theory of prefrontal cortex function. Annu. Rev. Neurosci. 24, 167–202. doi: 10.1146/annurev.neuro.24.1.167, 11283309

[ref29] Peters A. McEwen B. S. Friston K. (2017). Uncertainty and stress: why it causes diseases and how it is mastered by the brain. Prog. Neurobiol. 156, 164–188. doi: 10.1016/j.pneurobio.2017.05.004 28576664

[ref30] Pincus D. Metten A. (2010). Nonlinear dynamics in biopsychosocial resilience. Nonlinear Dyn. Psychol. Life Sci. 14, 353–380. 20887686

[ref31] Robinaugh D. J. Hoekstra R. H. A. Toner E. R. Borsboom D. (2020). The network approach to psychopathology: a review of the literature 2008–2018 and an agenda for future research. Psychol. Med. 50, 353–366. doi: 10.1017/S0033291719003404 PMC7334828 31875792

[ref32] Seth A. K. (2013). Interoceptive inference and emotion. Trends Cogn. Sci. 17, 565–573. doi: 10.1016/j.tics.2013.09.007, 24126130

[ref33] Stinson L. Liu Y. Dallery J. (2022). Ecological momentary assessment: a systematic review of validity research. Perspect. Behav. Sci. 45, 469–493. doi: 10.1007/s40614-022-00339-w, 35719870 PMC9163273

[ref34] Thayer J. F. Lane R. D. (2000). A model of neurovisceral integration in emotion regulation. J. Affect. Disord. 61, 201–216. doi: 10.1016/S0165-0327(00)00338-4, 11163422

[ref35] van der Maas H. L. J. Dolan C. V. Grasman R. P. P. P. Wicherts J. M. Huizenga H. M. Raijmakers M. E. J. (2006). A dynamical model of general intelligence: the positive manifold of intelligence by mutualism. Psychol. Rev. 113, 842–861. doi: 10.1037/0033-295X.113.4.842 17014305

[ref36] Wright A. G. C. Woods W. C. (2020). Personalized models of psychopathology. Annu. Rev. Clin. Psychol. 16, 49–74. doi: 10.1146/annurev-clinpsy-102419-125032, 32070120

[ref37] Wrzus C. Neubauer A. B. (2023). Ecological momentary assessment: a meta-analysis on designs, samples, and compliance across research fields. Assessment 30, 825–846. doi: 10.1177/10731911211067538, 35016567 PMC9999286

